# Two sides of the same coin: the intertwined relationship between photosynthesis and light stresses

**DOI:** 10.3389/fpls.2026.1958982

**Published:** 2026-10-26

**Authors:** Benny Jian Rong Sng, Ian Kin Yuen Choi, Sing Hui Leong, In-Cheol Jang

**Affiliations:** 1 Temasek Life Sciences Laboratory, National University of Singapore, Singapore, Singapore; 2 Disruptive & Sustainable Technologies for Agricultural Precision, Singapore-MIT Alliance for Research and Technology, Singapore, Singapore; 3 Department of Biological Sciences, National University of Singapore, Singapore, Singapore

**Keywords:** high-light stress, light signaling, light spectrum, photoreceptor, photosynthesis, photosynthetic pigment, shade avoidance response

## Abstract

Light is a crucial factor in plant growth, development, and productivity. While light primarily serves as the energy source to drive photosynthesis, environmental light fluctuations like vegetative shade and high-light intensity also function as signaling cues that regulate plant morphology, physiology and development. Under vegetative shade, the depletion of red and blue wavelengths reduces photosynthetic rate and triggers light signaling pathways that cause shade avoidance response (SAR). SAR promotes elongation growth and reduces photosynthetic pigment content, thereby compromising overall photosynthetic efficiency. In contrast, high-light stress occurs when light exposure exceeds the capacity of the photosynthetic apparatus, resulting in chlorophyll overexcitation, excessive production of reactive oxygen species (ROS), and subsequent oxidative damage and photoinhibition. To mitigate high-light stress, plants have developed diverse photoprotective mechanisms such as leaf and chloroplast movements, non-photochemical quenching of overexcited chlorophylls, ROS scavenging, and ATP/NADPH homeostasis. This review summarizes the molecular and physiological responses of plants to shade and high-light stress, with a particular focus on their impacts on photosynthesis. We further discuss current approaches for managing light stresses and optimizing photosynthetic performance in agriculture.

## 1 Introduction

As photoautotrophs, plants depend on light as an energy source to drive carbon fixation during photosynthesis. In the light reaction of photosynthesis, photosynthetic pigments absorb visible light ranging from 400 to 700 nm (photosynthetically active radiation, PAR). The light absorbance spectrum is attributed to the absorption spectra of photosynthetic pigments located within the light harvesting complexes (LHCs) of the chloroplast thylakoid membrane (Mirkovic et al., 2017; **Table 1**). Chlorophyll *a* (Chl *a*) is arguably the most crucial photosynthetic pigment in plants, as it functions in the reaction center to drive electron transport chains of the light-dependent reaction (Simkin et al., 2022). As the most abundant chlorophyll in plants, Chl *a* acts as the principal light-harvesting pigment for photosynthesis, exhibiting absorption peaks at approximately 400–450 nm and 660–675 nm (Simkin et al., 2022; **Figure 1A**). Other photosynthetic pigments, such as Chl *b* and carotenoids, function as antenna pigments that capture light wavelengths outside the optimal absorption spectrum of Chl *a*, and transfer the collected excitation energy to the reaction center (Simkin et al., 2022; **Figure 1A**). The combined absorption of all photosynthetic pigments determines the absorptance and action spectra of the plant, which is characterized by the reduced efficiency in green and yellow wavelengths due to stronger absorption of blue and red wavelengths by chlorophylls and carotenoids (Ptushenko et al., 2020, **Figure 1B**).

**Table 1:** Localization of photosynthetic pigments and plant photoreceptors.

Light-absorbing molecules	Name	Active/inactive form	Localization	Reference
Photosynthetic pigment	Chl *a*		LHC, reaction center (thylakoid membrane of chloroplast)	Simkin et al., 2022
	Chl *b*		LHC (thylakoid membrane of chloroplast)	Simkin et al., 2022
	Carotenoids		LHC (thylakoid membrane of chloroplast)	Simkin et al., 2022
Photoreceptors	PHYs	Pr (inactive)	Cytosol	Huq et al., 2003
		Pfr (active)	Nucleus	Huq et al., 2003
	CRY1		Nucleus and cytoplasm	Aguida et al., 2024
	CRY2		Nucleus	Aguida et al., 2024
	PHOT1	Inactive	Plasma membrane, chloroplast outer envelope	Ishishita et al., 2020
		Active	Cytosol	Ishishita et al., 2020
	PHOT2	Inactive	Plasma membrane, chloroplast outer envelope	Ishishita et al., 2020
		Active	Golgi apparatus	Ishishita et al., 2020
	UVR8	Inactive	Cytosol	Fang et al., 2022
		Active	Nucleus	Fang et al., 2022

**Figure 1:**
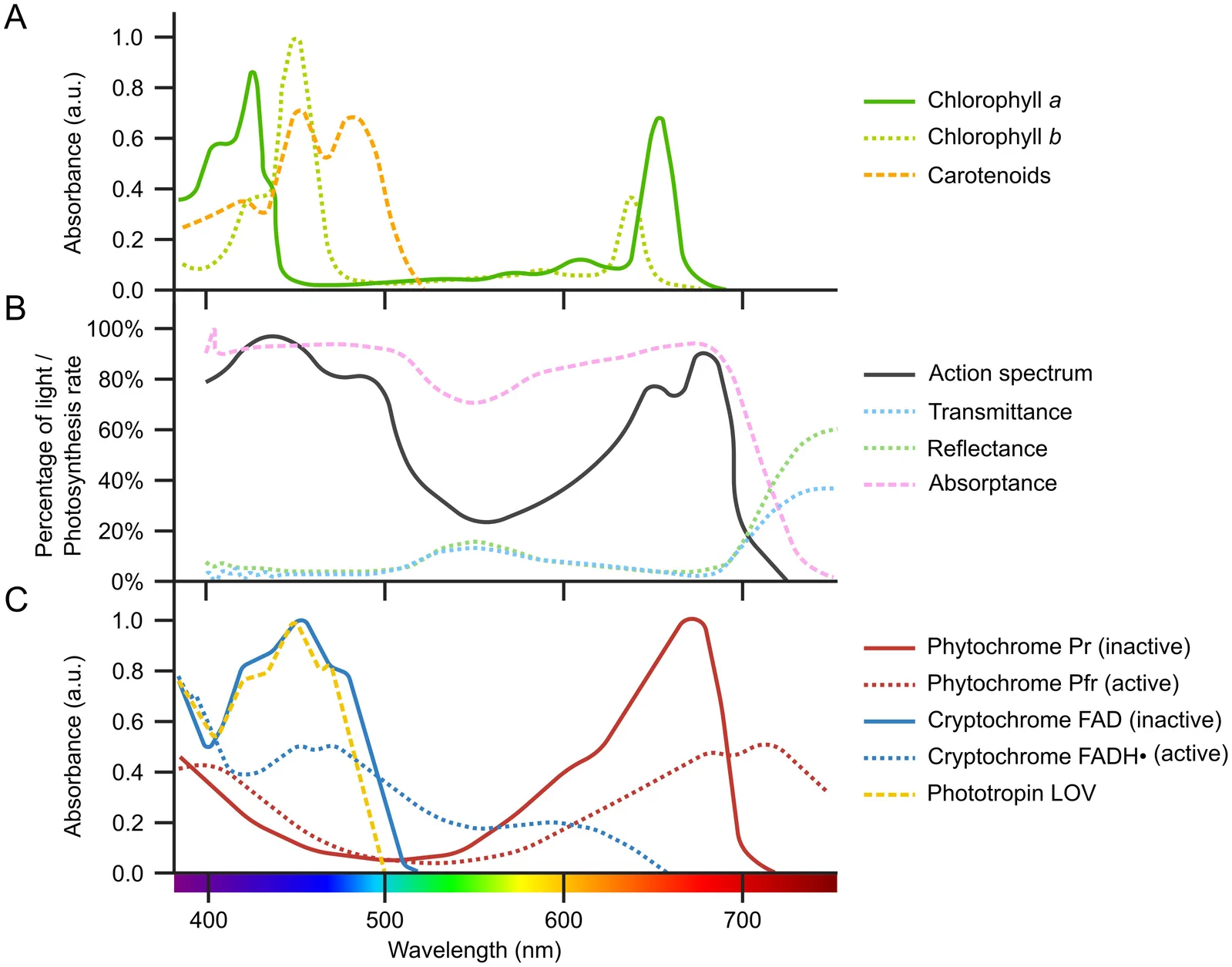
Comparison of the **(A)** absorbance spectra of photosynthetic pigments, **(B)** optical properties of plant tissue and action spectrum, and **(C)** absorbance spectra of photoreceptors. The absorbance spectrum of UVR8 is <400 nm. Absorbance is displayed in arbitrary units (a.u.). Graphs redrawn based on Campillo et al. (2012); Battle et al. (2020), and Liu and van Iersel (2021).

The absorbed light energy is used to drive photosynthetic electron transport chains. Excited electrons of the P680 chlorophyll in the reaction center of photosystem II (PS II) are transferred through a chain of acceptor molecules to the P700 chlorophyll in PS I (Stirbet et al., 2020). The transfer of electrons through PS II results in the movement of hydrogen ions from the thylakoid stroma to thylakoid lumen, resulting in a proton gradient across the thylakoid membrane that drives the flow of protons through ATP synthase to generate ATP (Stirbet et al., 2020). The electrons in PS I are further transferred through a series of acceptor molecules, which ultimately protonates NADP^+^ into NAPDH (Stirbet et al., 2020). ATP and NADPH are then used to drive the Calvin cycle, also known as the Calvin-Benson cycle or C3 cycle, for carbon fixation (Stirbet et al., 2020). Per cycle, carbon dioxide (CO_2_) reacts with three molecules of five-carbon ribulose 1,5-bisphosphate (RuBP) to form six molecules of the three-carbon 3-phosphoglyceric acid (PGA) (Sharkey, 2019). PGA is further converted to 1,3-diphosphoglycerate (DPGA), followed by glyceraldehyde-3-phosphate (GAP). For every six molecules of GAP, one molecule exits the Calvin cycle for the biosynthesis of sugars, while the remaining five molecules are recycled back into RuBP for a subsequent cycle (Sharkey, 2019).

Besides being a key driver of photosynthesis, light can also function as a signaling cue and stressor to plants. Plants grown under insufficient light quantity and quality, such as in vegetative shade, typically display undesirable growth and development that includes elongated stem and petiole tissues, weakened defense against pathogens, altered flowering time, and lower yield in crop plants (Carriedo et al., 2016). Vegetative shade is typically caused by neighboring vegetation absorbing red and blue wavelengths for photosynthesis, thereby resulting in a low light environment enriched in far-red and green wavelengths. On the other hand, excessive light can also be detrimental for plant growth. When light intensity surpasses the amount required for photosynthesis, the excess light energy can overexcite chlorophyll and induce the production of reactive oxygen species (ROS), leading to oxidative damage to plant cells.

This review explores plant responses to shade and high-light stress in the context of photosynthesis. We summarize the mechanisms underlying stress induction, signaling, and subsequent responses, along with their effects on photosynthetic capacity of the plant. Lastly, we briefly discuss how light stress can be managed in agriculture to maximize photosynthesis.

## 2 Shade avoidance response suppresses multiple aspects of photosynthesis

Sunlight provides a relatively uniform light intensity across the PAR spectrum, with lower intensities toward the blue wavelengths (<500 nm) (Sellaro et al., 2025). As red and blue wavelengths are preferentially absorbed for photosynthesis, green light penetrates deeper into leaf tissues, leaving a small proportion of green light to be reflected or transmitted through plant tissues (Ptushenko et al., 2020; Liu and van Iersel, 2021, **Figure 1B**). Far-red wavelengths are also readily transmitted through plant tissues, while ultraviolet (UV) light is largely absorbed by specialized photoreceptors for light signaling purposes (Rai et al., 2020; Liu and van Iersel, 2021; **Figure 1B**). As a result, the encroachment of neighboring vegetation results in a light condition known as vegetative shade, characterized by reduced red, blue, and UV wavelengths and increased green and far-red light (Franklin, 2008).

Light within the PAR spectral range is also readily absorbed by several families of photoreceptors in plants, enabling plants to perceive their environment and respond accordingly to light stresses. Most photoreceptors are holoproteins that are bound to light-responsive pigments called chromophores (de Melo and Alves, 2025). Absorption of specific light wavelengths induces a conformational change that activates photoreceptors, triggering downstream signaling cascades that ultimately modulate plant development and physiology (de Melo and Alves, 2025). Unlike photosynthetic pigments, photoreceptors are not confined within the chloroplasts and can localize to various subcellular compartments depending on their activation state (**Table 1**). Many photoreceptors accumulate in the nucleus upon activation, where they directly or indirectly regulate downstream transcription factors to coordinate light-responsive gene expression (**Table 1**).

Phytochromes (PHYs) are the primary photoreceptors responsible for sensing red and far-red wavelengths in plants (Paik and Huq, 2019). The inactive Pr form absorbs red light with maximal absorption at approximately 660 nm and converts into the biologically active Pfr form, which is subsequently deactivated upon absorbing far-red light with maximal absorption at approximately 730 nm (Li et al., 2011; **Figure 1C**). Interestingly, PHYA can also absorb and regulate plant responses to UV and blue light, albeit to a smaller extent (Chun et al., 2001). Cryptochromes (CRYs) predominantly mediate blue light signaling (Paik and Huq, 2019). Absorbing blue light (maximum absorption centered around 450 nm) generates the semireduced flavin radical (FADH·) in CRY, converting it into an active state (Sellaro et al., 2010; **Figure 1C**). In turn, the semireduced flavin exhibits increased absorbance in the green and yellow wavelengths (500–630 nm), where further photon absorption drives the flavin into an inactive, fully reduced state (FADH^-^) (Sellaro et al., 2010; **Figure 1C**). Phototropins (PHOTs) are another class of blue light photoreceptors that is responsible for directional light perception (Liscum et al., 2014). Upon absorbing blue light with peak absorption at 450 nm, PHOTs undergo autophosphorylation, activating the regulation of phototrophic response (Liscum et al., 2014; **Figure 1C**). Lastly, UV RESISTANCE LOCUS 8 (UVR8) specializes in the perception of UV-B light with peak absorption at approximately 295 nm, exhibiting no activity beyond 350 nm (Rai et al., 2020). Despite lacking an external chromophore, UVR8 utilizes the aromatic rings of several tryptophan residues to absorb UV-B light, which breaks the UVR8 homodimer and releases the active signaling monomer (Paik and Huq, 2019).

A comparison of the absorption spectra of plant photoreceptors (**Figure 1C**) highlights the important roles of red and blue wavelengths in light perception and signaling. Similar to light absorption for photosynthesis, photoreceptors exhibit reduced but non-null absorbance of green and yellow wavelengths (**Figure 1**). While the molecular mechanisms underlying red and blue light signaling have been extensively studied (Huq et al., 2024), green light signaling remains comparatively poorly understood, as no dedicated green light photoreceptor has yet been identified to date (Hao et al., 2023). The similarity between the absorbance spectra of photosynthetic pigments and photoreceptors suggests that because of the importance of red and blue wavelengths in photosynthesis, plants have developed specialized light-sensing mechanisms to monitor their light intensity and quality. Indeed, phytochromes, which originate in charophyte algae, are highly conserved across all land plants (Li et al., 2015). Moreover, while cryptochromes are found in both plants and animals, plant and algae cryptochromes are phylogenetically distinct from those in animals, highlighting separate evolutionary lineages (Haug et al., 2015).

Exposure to vegetative shade triggers SAR, which is a complex plant response that involves redirecting resources toward elongation growth to escape the competition of neighboring plants (Franklin, 2008). The regulation of SAR begins with photoreceptors (PHYs, CRYs, and UVR8) sensing changes in the far-red, red, blue, and UV light conditions (Fernández-Milmanda and Ballaré, 2021; **Figure 2**). These photoreceptors initiate signaling cascades with extensive crosstalk, cooperatively regulating downstream key players including the COP1/SPA complex (CONSTITUTIVELY PHOTOMORPHOGENIC 1/SUPPRESSOR OF PHYTOCHROME A-105), LONG HYPOCOTYL IN FAR-RED 1 (HFR1), and PHYTOCHROME INTERACTING FACTOR (PIF) family of transcription factors (Fernández-Milmanda and Ballaré, 2021; **Figure 2**). In turn, these transcription factors modulate the expression of downstream genes related to plant growth and development (Sng et al., 2023). Furthermore, light signaling pathways regulate the biosynthesis and homeostasis of phytohormones, most notably the auxin indole-3-acetic acid (IAA), which plays a central role in coordinating structural plant growth and development adaptations (Sng et al., 2023).

**Figure 2:**
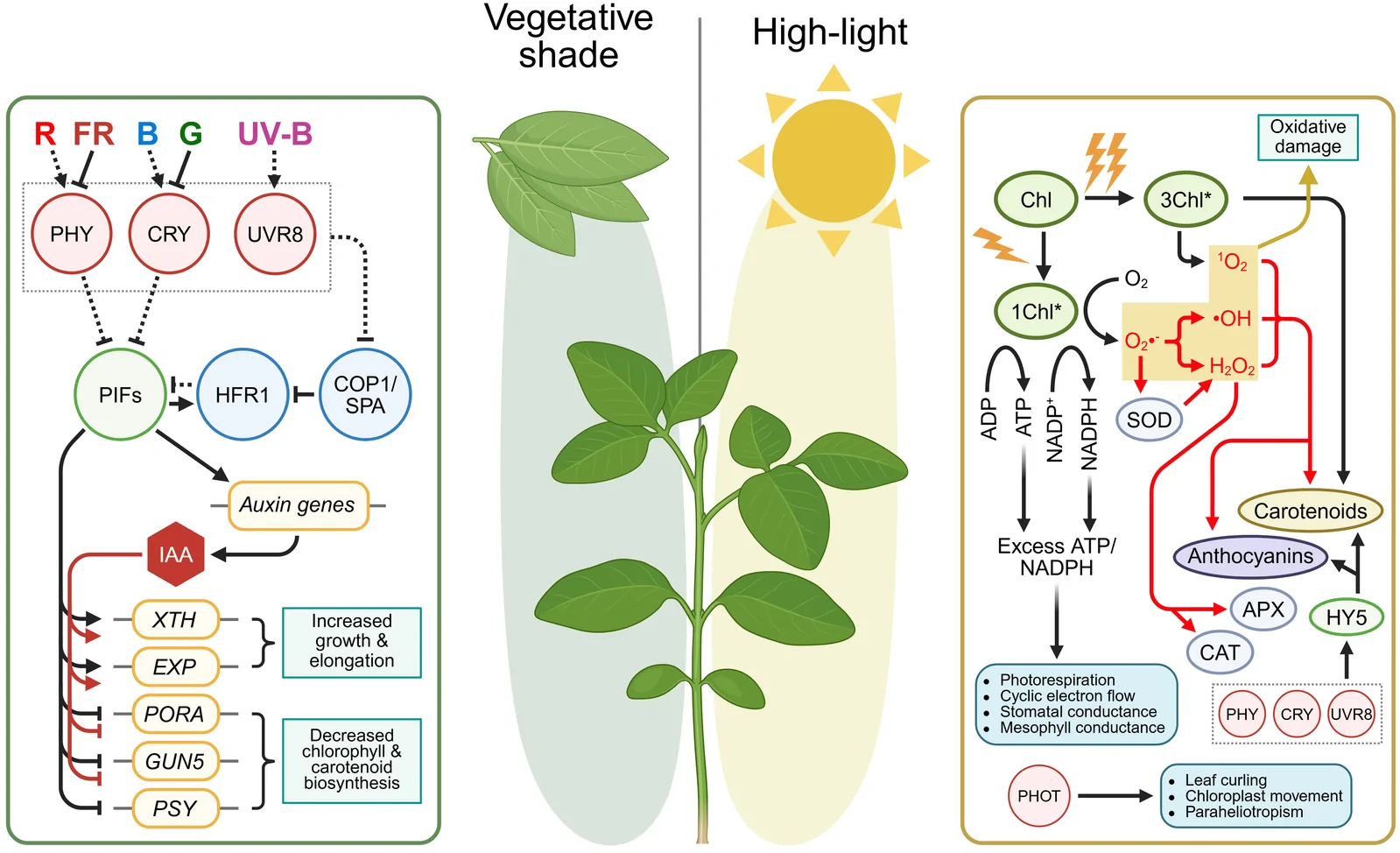
Absorption of incident light by neighboring plants depletes red, blue, and UV-B wavelengths beneath the leaf canopy, causing vegetative shade. Light that is depleted in red, blue, and UV-B wavelengths, but enriched in far-red and green wavelengths, results in the inactivation of photoreceptors (PHY, CRY, UVR8). This leads to downstream signaling such as the accumulation of PIFs and biosynthesis of phytohormone IAA. Together, they promote the expression of growth and elongation genes, while suppressing chlorophyll and carotenoid biosynthesis pathways, resulting in the overall development of shade avoidance response. Under high-light environment, excessive light absorption by chlorophyll (Chl) induces the production of reactive oxygen species (ROS), which causes oxidative damage in plants and an overall reduction in photosynthetic capacity. Plants have developed a variety of mechanisms to counter high-light stress. This includes non-photochemical quenching (NPQ) of overexcited Chl by carotenoids, ROS scavenging by antioxidants, and homeostasis of ATP and NADPH pools. Separately, light signaling photoreceptors and factors are also involved in high-light stress response, including phototropins (PHOT) for regulating plant and chloroplast movement and transcription factor HY5, which promotes the accumulation of antioxidant pigments carotenoids and anthocyanins. R, red light. B, blue light. G, green light. FR, far-red light. Photoreceptors (red circles): PHY, phytochrome; CRY, cryptochrome; UVR8, UV RESISTANCE LOCUS 8; PHOT, phototropin. Signaling intermediates (blue circles): HFR1, LONG HYPOCOTYL IN FAR-RED 1; COP1/SPA: CONSTITUTIVELY PHOTOMORPHOGENIC 1/SUPPRESSOR OF PHYTOCHROME A-105. Transcription factor (green circle): PIFs, PHYTOCHROME INTERACTING FACTORs; HY5, ELONGATED HYPOCOTYL 5. Yellow boxes (genes): *XTH*, *XYLOGLUCAN ENDO-TRANSGLYCOSYLASE/HYDROLASE*; *EXP*, *EXPANSIN*; *PORA*, *PROTOCHLOROPHYLLIDE REDUCTASE A*; *GUN5*, *GENOMES UNCOUPLED 5*; *PSY*, *PHYTOENE SYNTHASE*. IAA, Indole-3-acetic acid. Chlorophyll states (green ovals): 1Chl*, excited singlet chlorophyll state; 3Chl*, triple chlorophyll state. ROS species (red font): ^1^O_2_, singlet oxygen; O_2_•^-^, superoxide; H_2_O_2_, hydrogen peroxide; •OH, hydroxyl radical. Antioxidant enzymes (light blue ovals): SOD, superoxide dismutase; APX, ascorbate peroxidase; CAT, catalase. Created in https://BioRender.com.

SAR is characterized by a mix of phenotypic and physiological changes in the plant, including stem and petiole elongation, reduced leaf size and thickness, hyponasty, accelerated flowering and senescence, and diminished defense against pathogens (Franklin, 2008; Li et al., 2011; Wu et al., 2017; Li et al., 2023a; Sng et al., 2023; **Figure 2**). Although species- and cultivar-specific variations exist, these SARs are highly conserved across most plants, including crop species (Carriedo et al., 2016). A common response during shade avoidance is hyponasty, which results in the high petiole angles as the plant tries to reach upwards for more light (Michaud et al., 2017). However, this in turn reduces the top-down light interception on the leaves, due to their slanted angles (Falster and Westoby, 2003). In contrast, plants grown under optimal light condition develop large and flat leaves with a low petiole angle that maximizes light capture (Falster and Westoby, 2003). Shade also promotes the development of smaller and thinner leaves (Wu et al., 2017), thereby reducing the photosynthetically active surface area for capturing light and incomplete utilization of light for photosynthesis as it transmits through the leaf. At the molecular level, shade-induced PIFs upregulate the expression of cell wall modifying enzyme genes such as *XYLOGLUCAN ENDO-TRANSGLYCOSYLASE/HYDROLASE* (*XTHs*) and *EXPANSINs* (*EXPs*), which directly promote cell elongation (Zhang et al., 2013). Under shade, PIFs also enhance auxin biosynthesis, signaling and response (Sng et al., 2023). This further induces the expression of *XTHs* and *EXPs* with other cell wall expansion genes (Majda and Robert, 2018) and trigger hyponasty (Michaud et al., 2017).

Physiologically, the complex response of SAR is accompanied by extensive transcriptomic and metabolomic reprogramming (Sellaro et al., 2017; Zeng et al., 2026). PIFs suppress chlorophyll biosynthesis via their regulation of *REPRESSOR OF PHOTOSYNTHETIC GENES 1/2* (*RPGE1/2*) (Kim et al., 2023). In addition, PIFs directly inhibit carotenoid biosynthesis by repressing expression of *PHYTOENE SYNTHASE* (*PSY*), which encodes the rate-limiting enzyme in the pathway (Toledo-Ortiz et al., 2010). While shade enhances auxin-related responses, auxin signaling mediated by ARF7 and IAA14 also reduces chlorophyll content by suppressing the biosynthesis genes *PROTOCHLOROPHYLLIDE OXIDOREDUCTASE A* (*PORA*) and *GENOMES UNCOUPLED 5* (*GUN5*) (Luo et al., 2023). As such, SAR reduces photosynthetic pigments such as chlorophylls and carotenoids, which affect light harvesting for photosynthesis (Sng et al., 2020). SAR also lowers the expression of enzyme genes in the Calvin cycle, including *GLYCERALDEHYDE 3-PHOSPHATE DEHYDROGENASE* (*GAPDH*), *SEDOHEPTULOSE-1,7-BISPHOSPHATASE* (*SBPase*), *PHOSPHOGLYCERATE KINASE* (*PGK*), *FRUCTOSE-1,6-BISPHOSPHATASE* (*FBPase*), and *TRANSKETOLASE* (*TKT*) (Cagnola et al., 2012). While sucrose biosynthesis genes are down-regulated during SAR (Romero-Montepaone et al., 2021), genes related to starch production are also suppressed under shade, including starch precursor biosynthesis genes *OsSuS3* and *OsBT1* and starch biosynthesis enzymes *GBSSI* and *SSIII-2* in rice (Du et al., 2025). Besides altering gene expression, low light intensity also reduces the abundance of photosynthesis proteins, such as proteins involved in the electron transport chain, ATPase complex, and Calvin cycle (Liu et al., 2020). Together, these changes indicate that shade response suppresses multiple steps of photosynthesis, from the light-dependent reactions and carbon fixation to the subsequent biosynthesis of sugars.

## 3 Response to high-light stress safeguards photosynthetic capacity in plants

Under high-light intensity, the photosynthetic apparatus is exposed to excessive light energy that overloads its photosynthetic capacity, leading to the generation of ROS. As the captured energy exceeds the capacity of the Calvin cycle, overexcitation of chlorophyll can promote the formation of triplet chlorophyll, which readily reacts with triplet oxygen (^3^O_2_) to generate singlet oxygen (^1^O_2_) (Latowski et al., 2011; **Figure 2**). Moreover, over-conversion of available NADP^+^ to NADPH triggers the production of superoxide (O_2_•^-^) via the Mehler’s reaction, which can be further converted to hydrogen peroxide (H_2_O_2_) and hydroxyl radical (•OH) (Latowski et al., 2011; **Figure 2**). The accumulation of these ROS (^1^O_2_, O_2_•^-^, H_2_O_2,_ and •OH) causes photoinhibition of PS II and permanent damage to PS I, resulting in oxidative damage to other cellular components and thereby suppressing the overall photosynthetic rate (Sharma et al., 2023).

To prevent photoinhibition and oxidative stress, plants have evolved multiple mechanisms such as non-photochemical quenching (NPQ) and photoprotective pigment responses, which either reduce the amount of light absorbed by the photosynthetic apparatus or dissipate the excess energy as heat (**Figure 2**). High-light also results in responses such as leaf curling (Zhang et al., 2021), which is regulated by the photoreceptor PHOTs (Legris, 2023). PHOT signaling proteins PHYTOCHROME KINASE SUBSTRATE 3 (PKS3) and ROOT PHOTOTROPISM 2 (RPT2) have also been implicated in the regulation of leaf curling (Legris, 2023). Moreover, high-light triggers paraheliotropism in diverse plant species including rice, common bean, and tobacco, during which leaf angles are elevated to become parallel to the light rays, thereby minimizing direct light exposure and prevents photoinhibition (Zeng et al., 2022; Zhang et al., 2024). While the mechanism has not been extensively studied, paraheliotropism has been suggested to be related to water potential in the plant (Yu and Berg, 1994). High-light also induces the movement and redistribution of chloroplasts from periclinal walls to anticlinal walls of leaf cells, to reduce their total surface area exposed to light as well as to limit excess light absorption (Gotoh et al., 2018). The movement of chloroplasts under high-light is likewise regulated by PHOTs, which trigger the polymerization of chloroplast-actin filaments that facilitate chloroplast movement (Gotoh et al., 2018).

The photosynthetic apparatus also established several NPQ mechanisms to dissipate energy from overexcited chlorophylls. Through thermal deactivation, carotenoids absorb the excitation energy from chlorophylls and convert it into heat for dissipation (Ramel et al., 2012). Carotenoids also play an important role in the xanthophyll cycle for reducing oxidative stress caused by high-light. Zeaxanthin, antheraxanthin, and violaxanthin are xanthophylls that are derived from β-carotene in the carotenoid biosynthesis pathway (Fernández-Marín et al., 2021). While the epoxidation of zeaxanthin results in the biosynthesis of antheraxanthin and violaxanthin, high-light stress drives the de-epoxidation of violaxanthin to zeaxanthin, thereby utilizing the additional excitation energy (Fernández-Marín et al., 2021). In addition, H_2_O_2_ contributes to the maintenance of high zeaxanthin content under high-light condition by inhibition of ZEAXANTHIN EPOXIDASE (Kim et al., 2025). Besides carotenoids, cyclic electron flow in PS I also acts as another important photoprotective mechanism. Under normal light intensity, an excited P700 functions as the primary electron donor and transfers its electron through the electron transport chain to the final acceptor NADP^+^ (Stirbet et al., 2020). However, as high-light results in over-protonation of NADP^+^, electrons are transferred from reduced ferrodoxin in PS I back to plastoquinone in PS II, thus preventing further production of NADPH (Shikanai, 2014). Subsequently, electron transfer from reduced plastoquinone to cytochrome b_6_f complex drives proton pumping into the thylakoid lumen, promoting further production of ATP (Shikanai, 2014).

Excess energy from high-light is also dissipated by alternative pathways. While the Calvin cycle utilizes ATP and NADPH for carbon fixation and the production of glucose, photorespiration wrongly fixes oxygen into RuBP, resulting in synthesis of the side-product 2-phosphoglycolate (Hagemann and Bauwe, 2016). As 2-phosphoglycolate cannot be used to synthesize glucose, it undergoes multi-step conversion involving transport through the peroxisome and mitochondrion, ultimately producing carbon dioxide, ammonia, and PGA that is recycled back into the Calvin cycle (Hagemann and Bauwe, 2016). Although photorespiration does not produce any sugar, it consumes ATP and NADPH generated by the light-dependent reaction. Thus, photorespiration prevents over-accumulation of ATP and NADPH under high-light (Wingler et al., 2000). Enriching the overall photosynthetic rate also alleviates the high amounts of excitation energy from the light reaction. Stomatal opening and conductance increase under elevated red and blue light intensities, thus enhancing the influx of carbon dioxide and efflux of oxygen to maximize photosynthesis (Yang et al., 2020a). In a similar manner, mesophyll conductance increases in Eucalyptus, *Nicotiana tabacum*, Camellia, and Rice under high-light, allowing more gas exchange for photosynthesis (Yang et al., 2020b).

As high-light triggers the overproduction of ROS in plants, antioxidants play a prominent role in nullifying ROS, thus protecting the plant against oxidative stress and cell damage (Moustaka and Moustakas, 2025). The enzymatic antioxidants include superoxide dismutase (SOD), ascorbate peroxidase (APX), and catalase (CAT), all of which demonstrate up-regulated expression (Filiz et al., 2019) and elevated activity under high-light stress (Lall et al., 1999). While SOD functions in reacting O_2_•^-^ into H_2_O_2_ and oxygen, APX and CAT further reduce H_2_O_2_ into water (Filiz et al., 2019; Moustaka and Moustakas, 2025; **Figure 2**). ROS are also quenched by non-enzymatic pigments such as carotenoids and anthocyanins. Carotenoids scavenge ROS to form carotenoid radicals that are further reacted with ascorbate to regenerate the ROS-scavenging carotenoids (Sandmann, 2019). While carotenoids are located in the thylakoid membrane of chloroplasts, anthocyanins are stored in the central vacuole (Zheng et al., 2021). Although anthocyanins are not in spatial proximity to the main sources of ROS in plants, several studies have shown that anthocyanins can confer photoprotection by scavenging ROS like O_2_•^-^ and H_2_O_2_ (Xu et al., 2017; Zheng et al., 2021). Furthermore, their characteristic dark-purple coloration in plants confers additional photoprotection by filtering out excess light (Zheng et al., 2021). Importantly, high-light increases the abundance of carotenoids and anthocyanins in plants (Balcke et al., 2024). This could be partly regulated by photoreceptors CRYs, PHYs, and UVR8 as well as the COP1/SPA complex, which promote light signaling transcription factors ELONGATED HYPOCOTYL 5 (HY5) and B-BOX ZINC FINGER PROTEIN (BBX) 20/21/22 that activate the anthocyanin biosynthetic pathway (Araguirang and Richter, 2022). HY5 also triggers the expression of *ABA INSENSITIVE 5* (*ABI5*), which stimulates RESPIRATORY BURST OXIDASE HOMOLOG 1 (RBOH1)-dependent ROS production and activates a strong antioxidant response that confers further photoprotection (Wang et al., 2018). Furthermore, HY5 has been reported to regulate ROS levels via a CRY1-HY5-MYB signaling cascade (Chang et al., 2025). This suggests that HY5 coordinates light signaling, anthocyanin biosynthesis, and oxidative stress responses. HY5 also promotes the expression of *PSY*, which is the first dedicated step in carotenoid biosynthesis (Kim and Eom, 2025). High-light stress also promotes anthocyanin biosynthesis through other transcription factors besides HY5. In apples, high-light activates MdWRKY1, which promotes the expression of long noncoding RNA (lncRNA) *MdLNC499*, followed by MdERF109 to ultimately induce anthocyanin biosynthesis (Ma et al., 2021). While high-light stress triggers multiple response mechanisms in plants, they collectively confer protection against photodamage and oxidative damage, thereby safeguarding the photosynthetic capacity of the plant.

## 4 Managing light stresses and optimizing photosynthesis in agriculture

The ultimate goal of increasing photosynthesis in agriculture is primarily to improve plant growth and maximize crop biomass and yield. Technological advancements in agriculture such as automated supplemental lighting (Pereira and Glória Gomes, 2025) and spectral manipulation using colored shade nets (Kotilainen et al., 2018) can allow for greater control over light intensity and wavelengths, thereby improving light stress response and photosynthetic rates, ultimately contributing to higher crop yields.

In open-field agriculture, fluctuating light intensities and spectra create more complex light environments where plants experience sudden and transient shifts to shade or high-light stress. In fluctuating shade that is punctuated by repeated sunflecks, photoreceptors UVR8 and CRY1 become more sensitive and easily activated, resulting in lower PIF4 and PIF5 abundance and thus greater suppression of SAR (Sellaro et al., 2025). In addition, longer duration of light exposure increases HY5 gene expression and protein abundance in the nucleus, thereby further repressing PIF-dependent shade response (Sellaro et al., 2025). In contrast, protection against fluctuating high-light stress is dependent on PROTON GRADIENT REGULATION 5 (PGR5), which facilitates cyclic electron flow in PS I (Allahverdiyeva et al., 2015). Responses to high-light fluctuations are also regulated by STATE TRANSITION 7 (STN7) and thylakoid lumen protein TLP18.3 that are essential for maintaining and repairing PS II function (Allahverdiyeva et al., 2015).

To manage excessive vegetative shading, recent modeling methods predict that plant canopies with upright leaves at the upper canopy and horizontal leaves in the lower canopy offer the highest light use efficiency under top-down lighting (Slattery and Ort, 2021). This can be seen in the breeding of superior rice hybrids that have these attributes (Slattery and Ort, 2021). Likewise, maize was developed to have a smart-canopy architecture that minimizes self-shading, with high petiole angles in the upper leaves and low angles in the bottom leaves (Tian et al., 2024). In addition, the effects of shade response can be inhibited by developing *pif* knockout mutations in plants, which results in a more compact plant structure (Kunta et al., 2025).

Improving photosynthetic capacity under high-light stress can be achieved by targeting various aspects of the photosynthetic process, including enhancing light capture, absorption and conversion, recovery of NPQ, and optimization of the Calvin cycle (Li et al., 2023b). For example, reducing the antenna size of light harvesting complexes improved photosynthetic rate and lowered NPQ (Li et al., 2023b). Moreover, mutation in the upstream regulatory gene *HIGH PHOTOSYNTHETIC EFFICIENCY 1* (*HPE1*) increases chlorophyll *a/b* ratio and thereby enhances photosynthetic efficiency (Jin et al., 2016). Optimization of the Calvin cycle typically involves improving the efficiency of RIBULOSE-1,5-BISPHOSPHATE CARBOXYLASE/OXYGENASE (RuBisCO), which is known as being a rate-limiting enzyme in carbon fixation due to its poor catalytic rate (Li et al., 2023b). Engineering of the photorespiratory pathway for improved photosynthetic capacity under stress is also a viable strategy (Sun et al., 2024).

Furthermore, as light stress typically invokes metabolite changes, some key metabolites are potentially useful in monitoring for signs of light stress. For example, as IAA is a key phytohormone that drives SAR, technologies for real-time monitoring of IAA may help to alleviate the development of shade phenotypes (Khong et al., 2025). In a similar manner, high-light stress can also be detected by monitoring for changes in H_2_O_2_ and phytohormone salicylic acid (Ang et al., 2024). Raman spectroscopy coupled with machine learning algorithm has also been employed for the diagnosis of SAR and high-light stress (Sng et al., 2020; Patil et al., 2025). These diagnostic technologies can allow for early mitigation of light stress in plants as a species-independent tool, thereby ensuring that the crops can achieve maximum photosynthetic capacity throughout their growth cycle and biomass production.

## 5 Conclusion

In summary, plant responses to light stresses inevitably affect photosynthesis. While SAR prioritizes elongation growth over maintenance of photosynthesis, high-light stress triggers multiple mechanisms to prevent extensive damage to the photosynthetic apparatus. Although SAR is primarily regulated by photoreceptors and light signaling pathways, plant responses to high-light stress involve the dissipation of excess energy, ROS scavenging, homeostasis of photosynthesis components, and regulation by light signaling factors. Understanding how light stresses regulate plant physiology and photosynthetic capacity are important for implementing strategies to overcome them in agriculture and to maximize photosynthesis even under unfavorable light conditions.
